# Sentinel events and predictors of suicide among inpatients at psychiatric hospitals

**DOI:** 10.1186/1744-859X-11-4

**Published:** 2012-02-16

**Authors:** Yi-Lung Chen, Dong-Sheng Tzeng, Ting-Sheng Cheng, Chien-Hung Lin

**Affiliations:** 1 Department of Psychiatry, Kaohsiung Armed Force General Hospital, Kaohsiung, Taiwan; 2 Hyperbaric and Undersea Institute, National Defense Medical Center, Taipei, Taiwan; 3 Calo Psychiatric Center, Pingtung County, Taiwan

**Keywords:** retrospective study, sentinel event, suicide

## Abstract

**Background:**

The objective of this study was to assess sentinel event analysis and relative factors in different mental healthcare settings. In addition, the occurrence of sentinel events in different hospital settings was compared and potential risk factors contributing to sentinel events identified.

**Methods:**

A total of 75 consecutive adult subjects were enrolled from 2 psychiatric units, 1 within a general hospital and 1 at a psychiatric hospital in southern Taiwan. A retrospective chart review of the psychiatric inpatients was conducted for patients that met the criteria for a sentinel event between July 2004 and May 2011. A comparison of the hospital settings was made and differences between suicidal and non-suicidal sentinel events studied.

**Results:**

Psychiatric patients that received general hospital psychiatric services (1) appeared to experience a sentinel event soon after admission, (2) the time between the sentinel event occurrence and patient death was shorter, (3) there was a higher probability of potential medical illness than among inpatients treated at a specialized psychiatric hospital, (4) the sentinel event subjects that committed suicide were younger, had a shorter hospital stay, shorter time to occurrence of the sentinel event followed by an unexpected death than the non-suicidal group, and (5) a younger age, higher education level, previous suicide attempt and family psychiatric history were important predictors of suicide among psychiatric inpatients.

**Conclusions:**

The results of this study suggest that psychiatric inpatients treated at a general hospital require careful examination for potential physical illness and greater efforts to prevent suicide. A younger age, higher education level, history of a previous suicide attempt and family psychiatric history are additional risk factors for suicide among these patients.

## Introduction

In order to improve patient safety, there have been significant changes in the way health services are provided in the hospital setting [1]. The delivery of mental health services is very complicated; it requires action at all levels of the healthcare organization that is coordinated and integrated in accordance with the principles of clinical governance [1].

The terms 'sentinel event' and 'medical error' are not synonymous; not all sentinel events occur because of an error and not all errors result in sentinel events. The Joint Commission on Accreditation of Healthcare Organizations (JCAHO, now the Joint Commission) updated their definition of a sentinel event in 2007; the current definition is as follows: a sentinel event is an 'unexpected occurrence involving death or serious physical or psychological injury, or the risk thereof'. The phrase 'or the risk thereof' includes any variation for which a recurrence would carry a significant chance of a serious adverse outcome. Such events are called 'sentinel' because they signal the need for immediate investigation and response [2].

Many medical and health service managers have implemented a sentinel event policy to strengthen the reporting, monitoring, and management of major incidents. Such a policy must be implemented in accordance with the underlying healthcare system, human activities, work processes, and technical factors that may contribute to the occurrence of sentinel events [3].

Suicide occurrence in a hospital setting is one of the most commonly reported adverse events (12.4%), second only to surgical errors; about half (52%) of the suicides occur in psychiatric wards [4]. Previous reports have indicated that the range of inpatient suicide is 5 to 15 and 100 to 400 every 100,000 admissions to psychiatric and general hospitals, respectively. Patient characteristics such as male gender, depressive disorders, violent behavior, history of previous suicide attempts, absence without official leave and the rapid occurrence of suicide after admission are known risk factors for inpatient suicide in psychiatric or general hospitals [5-14].

A review of the current published literature shows a lack of data on comparison of sentinel events between admissions to psychiatric hospitals and admissions to psychiatric units in general hospitals; little is known about the associated suicidal characteristics and predictors of suicide among these inpatients, especially in Taiwan. The primary goal of this study was to assess sentinel event reporting in different mental healthcare settings. In addition, the occurrence of a sentinel event at one psychiatric hospital was compared to one psychiatric unit in a general hospital in southern Taiwan by retrospective chart review (between July 2004 and July 2011) to identify potential risk factors contributing to the sentinel event.

## Methods

### Participants

The redesigned care network model was used to recruit consecutive adults from two hospital settings, a psychiatric hospital and a psychiatric unit in a general hospital, in southern Taiwan. A network of mental health services has been created by coordinating services at a general hospital, a psychiatric hospital, 10 outpatient clinics, and community programs [15]. Inpatients that met the criteria of a sentinel event, according to the definition reported by JCAHO were recruited for this study. The criteria for the psychiatric diagnosis of the subjects was based on the *Diagnostic and Statistical Manual of Mental Disorders, Fourth Edition *(DSM-IV).

### Measurements

The hospital's institutional review board approved this study. A retrospective chart review of the psychiatric inpatients that met the definition of a sentinel event was performed between July 2004 and May 2011. The demographics for all patients were reviewed. Patients that committed suicide were defined as cases and compared to other sentinel events. The definition of a sentinel event not associated with suicide was an unexpected sentinel event not associated with suicide.

The variables that were compared included: gender, age, disease duration, level of education, smoking, alcohol use, drug misuse, medical history, previous suicidal history, committed suicide or not, hospital stay before occurrence of the sentinel event, the time of occurrence of a sentinel event followed by an unexpected death, a result of death or serious disability, transfer to the intensive care unit (ICU) or not, nursing care time and the seniority of attending doctors. In addition, patients that experienced a sentinel event were categorized as suicidal or non-suicidal for comparison of characteristics.

### Statistical analysis

SPSS V.15.0 (SPSS Inc., Chicago, IL, USA) was used for the demographic, descriptive, exploratory χ^2 ^analyses. A Student's t test was carried out to compare the differences between patients in the psychiatric unit of a general hospital and those at a psychiatric hospital to determine whether there were significant differences. For further analysis, a logistic regression model was used to identify important predictors of suicide after a sentinel event.

## Results

A total of 75 sentinel events were reported from the 2 hospital settings studied; 52 patients were from a psychiatric unit within a general hospital and 23 patients were from a psychiatric hospital. Among them, schizophrenia was the most common diagnosis (n = 38, 50.67%), followed by bipolar disorder (n = 18, 24%), and major depressive disorder (n = 7, 9.33%), substance-related disorder (n = 6, 8%), depressive disorder (n = 3, 4%), and dementia (n = 3, 4%). The comparison of demographic data between two different psychiatric mental healthcare settings is shown in Table 1.

**Table 1:** The χ^2 ^and t test results of patients with a sentinel event in general hospital and psychiatric hospital settings

Characteristic	General hospital (N = 52)	Psychiatric hospital (N = 23)	*P *value
Age	45.6 ± 17.6	45.9 ± 14.5	0.282
Disease duration, years	11.5 ± 9.7	12.9 ± 7.2	0.405
Inpatient episode	3.9 ± 3.4	4.4 ± 4.2	0.656
Length of hospital stay before sentinel event occurrence, h	52.7 ± 65.6	152.9 ± 206.8	0.001
Level of education, years	9.4 ± 3.9	9.0 ± 3.2	0.280
Time between sentinel event occurrence and resulting death, h	5.5 ± 14.4	21.9 ± 80.9	0.011
Nursing care, h/day	2.2 ± 0.8	2.0 ± 1.4	0.234
Seniority of attending doctors, years	9.5 ± 6.3	7.4 ± 5.4	0.212
Male sex	28 (53.8%)	16 (69.6%)	0.202
Female sex	24 (46.2%)	7 (30.4%)	
Committed suicide	19 (36.6%)	9 (39.1%)	0.831
Non-suicide	33 (63.4%)	14 (60.9%)	
Resulted in death	43 (82.7%)	21 (91.3%)	0.331
Serious disability	9 (17.3%)	2 (8.7%)	
Referral to ICU	15 (28.8%)	5 (21.7%)	0.521
No referral to ICU	37 (71.2%)	18 (78.3%)	
Smoker	24 (46.2%)	14 (60.9%)	0.2408
Non-smoker	28 (53.8%)	9 (39.1%)	
Drug misuse history	8 (15.4%)	2 (8.7%)	0.662
No drug misuse history	44 (84.6%)	21 (91.3%)	
Alcohol misuse history	13 (25%)	8 (34.8%)	0.479
No alcohol misuse history	39 (75%)	15 (65.2%)	
Relevant medical history	30 (58.8%)	6 (26.1%)	0.009
No relevant medical history	21 (41.2%)	17 (73.9%)	
Suicidal history	20 (38.5%)	6 (26.1%)	0.299
No suicidal history	32 (61.5%)	17 (73.9%)	

Data are given as mean ± SD or N (%).

ICU = intensive care unit.

There were three demographic variables compared between the two different hospital settings found to have a significant difference. Firstly, the mean occurrence time of the sentinel event after admission for psychiatric inpatients from the general hospital was 52.7 ± 65.6 h, and for patients from the psychiatric hospital was 152.9 ± 206.8 h. Secondly, the mean occurrence time of a sentinel event followed by an unexpected death for psychiatric inpatients from the general hospital was 5.5 ± 14.4 h, and for inpatients from the psychiatric hospital was 21.9 ± 80.9 h. Thirdly, the mean nursing time for psychiatric inpatients from the general hospital was 2.2 ± 0.8 h/day, and for inpatients from the psychiatric hospital was 2.0 ± 1.4 h/day. There was no statistically significant difference found for the other demographic variables with regard to the sentinel events in the two different hospital settings based on the χ^2 ^analysis and t tests as shown in Table 1.

Comparison of the sentinel events between the suicidal and non-suicidal patients showed that 29 patients that committed suicide were assigned to the suicidal group and the other patients (n = 46) were assigned to the non-suicidal group. Comparison of the demographic data between the suicidal and non-suicidal group is shown in Table 2.

**Table 2:** The independent t test in patients with a sentinel event by suicidal and non-suicidal groups

Characteristic	Suicidal group (N = 29)	Non-suicidal group (N = 46)	*P *value
Age	39.3 ± 11.6	49.7 ± 18.0	0.015
Disease duration, years	9.9 ± 8.3	13.1 ± 9.2	0.813
Inpatient episode	5.3 ± 4.1	3.3 ± 3.1	0.225
Length of hospital stay before sentinel event occurrence, h	109.1 ± 158.4	47.3 ± 61.2	0.001
Level of education, years	10.4 ± 3.9	8.6 ± 3.5	0.319
Time between sentinel event occurrence and resulting death, h	4.4 ± 10.1	14.7 ± 59.7	0.029
Nursing care, h	1.8 ± 1.1	2.4 ± 0.9	0.521
Seniority of attending doctors, years	9.5 ± 5.9	8.4 ± 6.2	0.472

Values are mean ± SD.

Two of the demographic variables showed a significant difference. The mean age of the suicidal group was 39.3 ± 11.6 years, and 49.7 ± 18.0 years for the non-suicidal group. Hospital stay after admission for the suicidal group was 109.1 ± 158.4 h, and for the non-suicidal group was 47.3 ± 61.2 h. The time between the occurrence of the sentinel event and death was 109.1 ± 158.4 h for the suicidal group, and 47.3 ± 61.2 h for the non-suicidal group. The other demographic variables showed no significant differences with regard to sentinel events when compared between the suicide and non-suicide groups.

A multivariate regression analysis was performed to explore the relative factors associated with suicide among the 29 sentinel events followed by suicide; the significant predictors of suicide were: a younger age (B = -0.062; *P *value = 0.027), higher education level (B = 0.256; *P *value = 0.022), previous suicide history (B = 2.519; *P *value = 0.001), and family psychiatric history (B = 2.932; *P *value = 0.002) (see Table 3).

**Table 3:** Multivariate analysis of the predictors of suicidal sentinel event by logistic regression model

Predictor variable	B	SE	Wald χ^2^	95% CI	*P *value
Age	-0.062	0.028	4.915	0.89 to 0.99	0.027
Education level	0.256	0.112	5.275	1.04 to 1.61	0.022
Previous suicide history	2.519	0.721	12.204	3.02 to 51.00	0.001
Family psychiatric history	2.932	0.947	9.592	2.93 to 119.96	0.002

## Discussion

The chart review performed in this study compared any potential contributing factors associated with the sentinel event in two different mental healthcare facilities. The main findings of this study were the following: inpatients that received psychiatric services in a general hospital appeared to have a shorter duration between the time of admission and the sentinel event than that of inpatients treated at a psychiatric hospital. In this connection, both the time between the occurrence of the sentinel event that resulted in death in a general hospital was shorter and the probability of potential medical illness in a general hospital was higher than those of a psychiatric hospital. Furthermore, the study subjects in the suicidal sentinel event group were younger than those in the non-suicidal group. The duration between the time of admission and occurrence of the sentinel event in the suicidal group was shorter and the occurrence of a sentinel event followed by an unexpected death was shorter in the suicidal group. Finally, a younger age, higher education level, previous suicide attempt and family psychiatric history were significant important predictors of psychiatric inpatient suicide.

The findings of this study confirm that psychiatric inpatients in a general hospital were vulnerable to sentinel events soon after admission with serious outcomes including unexpected death and serious disability [5,13]. It is well known that psychiatric patients treated at acute care general hospitals present with more serious psychiatric symptoms and a higher probability of potential medical illness. These psychiatric inpatients with a medical history were not excluded from this retrospective chart review study. Although these patients received more nursing care, this did not reduce the occurrence of a sentinel event. This finding suggests that inpatients treated at a psychiatric unit within a general hospital should have a complete medical investigation including a careful physical examination. Identifying and treating underlying medical and psychiatric comorbidities must be part of the treatment plan to reduce the likelihood of recurrence.

In addition, this study compared the characteristics of patients in the suicide and non-suicide groups with regard to sentinel events. A previous study reported that timing is a risk factor for suicide in the hospital [4]. The period after hospital discharge or during home leave is when most suicides take place; a time when patients often decide to take their own life [4]. The retrospective chart review performed in this study showed similar results: 19 patients (65.5%) committed suicide outside of hospital and another 10 patients (34.5%) committed suicide inside the hospital. Moreover, the time of suicide after a sentinel event occurrence was soon after admission in the non-suicide group. Therefore, suicide prevention in recently admitted patients in the acute ward setting is critical for patient care.

The results of this study suggest that the key contributing factors associated with inpatient suicide (including home leave or hospital discharge) are age, education level, previous suicide attempt and family psychiatric history, apart from the underlying illness of the patient. There may be some other factors that contribute to suicide not as yet determined. One publication reported that in addition to physical environmental risk factors, systemic shortcomings with regard to care contribute to suicide, including inadequate screening and assessment, care planning and observation; insufficient staff orientation and training; poor staff communication; inadequate staffing; and lack of information about suicide prevention and referral resources [16]. With regard to our chart review study, these associated potential risk factors were not evaluated in detail as part of the chart review although we did not find differences in the seniority of the attending physician or the number of nursing hours. Therefore, associated epidemiological information should be recorded thoroughly and made available to healthcare managers and clinicians. The findings of this study support the need for improved systems and work efforts to minimize the recurrence of sentinel events associated with suicide.

The data compared between the suicidal and non-suicidal groups showed that the mean age and duration of mental illness, of the patients that committed suicide, were younger than in the group of non-suicidal patients. About half of the suicidal study subjects (19/29, 51.7%) had a past history of a suicide attempt. Previous episodes of self-abusive behavior and a history of suicide attempts, especially during the index admission, were also significant predictors [17-19]. The findings of this study are consistent with a previous report that showed that psychiatric inpatients with a history of suicidal behavior were mainly young patients with a previous suicide attempt [20]. Previous studies have demonstrated that younger patients were found to have higher rates of suicidal ideation or attempts than older age groups [21-24]. Although these study subjects were collected from the general population in the community. It is well known, based on retrospective survey data, that over 90% of suicides have a diagnosable mental illness [25,26]. The findings of this study confirm that young age is a risk factor for suicide following a sentinel event.

In contrast to earlier findings, however, there was no evidence of an association between the duration of mental illness and suicidal events [27,28]. Since the definition of a sentinel event is an unexpected occurrence involving death, physical or psychological injury, a possible explanation for this result is that some suicidal events are associated with less violent methods and cause minor physical injuries that may be underreported.

A logistic regression analysis was performed to identify the potential predictors of suicidal events associated with a sentinel event. The variables studied included age, gender, education level, marital status, and family psychiatric history. In addition to younger age and previous suicide attempt, other significant predictors identified were a higher education level and a family psychiatric history. A study demonstrated that a family history of a psychiatric disorder was strongly associated with psychiatric inpatient suicide [29]. A review of the currently published literature showed positive prior report of a correlation between education level and inpatient suicide [30]. One explanation might be the younger age of suicide cases with regard to education level compared to the non-suicidal group. Another explanation is the selective bias associated with a small sample size.

Another issue is that all study subjects were part of an integrated care model of psychiatric service, either in a psychiatric unit within a general hospital or a psychiatric hospital [15]. These two psychiatric hospital settings have the same integrated care model and provide an intensive care experience for their psychiatric patients. A suicide assessment protocol was thoroughly performed to identify subjects and the appropriate psychiatric services provided. This would include 24 hour monitoring, high levels of nursing care, routine assessment of mental symptoms and security screens in order to minimize the occurrence of a serious sentinel event. Since there is little data in the literature for comparison, different care models must be considered in the future.

This retrospective study had several limitations. The retrospective chart review methodology limited a more detailed recording of demographic information. The correlation of physical and mental illness with the reported sentinel event could not be clearly linked based on a chart review. Objective assessment tools should be presented on charts to help in this matter. The characteristics associated with suicidal and non-suicidal sentinel events, in patients admitted for integrated psychiatric services, may not be applicable to a traditional psychiatric care model. The relatively small sample size also causes wide 95% confidence intervals for the odds ratios, which make the predictors and estimates less reliable.

## Conclusions

Based on the comparative results of two different psychiatric hospital settings, hospitalized psychiatric inpatients treated at a general hospital appear to have unidentified critical medical conditions more frequently than the inpatients treated in a psychiatric hospital setting. The goal of this study was to identify factors that might help predict suicidal behavior associated with sentinel events. Such factors might help prevent suicide, and improve patient care.

## Competing interests

The authors declare that they have no competing interests.

## Authors' contributions

Dr. DT contributed to the conception and design of the study. Dr YC contributed to the data collection process and assembling of data. Dr. TC contributed to overlooking the data collection and data analysis. Dr. CL also contributed to the analysis of the data and further interpretation of the data. Dr. YC was involved in the overall research process. All authors contributed to the drafting of the manuscript and approved of the final manuscript.
